# What's that smell? A pictorial review of the olfactory pathways and imaging assessment of the myriad pathologies that can affect them

**DOI:** 10.1186/s13244-020-00951-x

**Published:** 2021-01-07

**Authors:** Geoffrey Lie, Alexander Wilson, Thomas Campion, Ashok Adams

**Affiliations:** Radiology Department, Royal London and St Bartholomew’s Hospital, Barts Health NHS Trust, London, UK

**Keywords:** Olfactory, Olfactory bulb, Olfactory tract, CT, MRI

## Abstract

The olfactory pathway is composed of peripheral sinonasal and central sensorineural components. The wide variety of different pathologies that can affect the olfactory pathway reflect this complex anatomical relationship. Localising olfactory pathology can present a challenge to the reporting radiologist. This imaging review will illustrate the normal anatomy of the olfactory system and describe a systematic approach to considering olfactory dysfunction. Key concepts in image interpretation will be demonstrated using examples of olfactory pathway pathologies.

## Key points

- Olfactory dysfunction is a prevalent and common complaint.
- The olfactory system is composed of peripheral and central components.
- CT provides assessment of peripheral sinonasal olfactory disease and bone-related pathology.
- MRI provides soft tissue characterisation and intracranial assessment.
- CT and MRI are complementary in characterising olfactory system lesions.

## Background

Olfactory dysfunction is a common symptom affecting approximately 20% of the general population [1]. Moreover, a significant number of patients presenting with loss of taste sensation have olfactory rather than gustatory deficits [2]. Olfactory dysfunction ranges from reduced smell detection (“hyposmia”) to complete loss of smell (“anosmia”). It also encompasses an abnormal sense of smell (“dysnomia”)—including loss of smell intensity (“parosmia”), inability to recognise smells (“cacosmia”) and olfactory hallucinations (“phantosmia”). Deficits in olfactory function can have a profound impact upon an individual’s overall quality of life and can also affect one’s ability to identify environmental hazards with important safety implications [3].

The olfactory nerves (Cranial Nerve I) and the olfactory pathways connect the peripheral nasal cavity with the central intracranial olfactory system. Olfactory dysfunction can be broadly divided into peripheral conductive and central sensorineural pathologies. Processes that impair the peripheral conduction of odour particles to the olfactory epithelium include sinonasal inflammatory disease and obstructive sinonasal mass lesions. Disruption of olfactory neuronal function and the central olfactory pathways can be secondary to a spectrum of postviral, traumatic, congenital, and neurodegenerative aetiologies. This article will review the normal anatomy of the olfactory pathways, the CT and MR imaging techniques available to image different components of the olfactory pathway, and highlight the spectrum of olfactory system pathologies.

## Functional anatomy and imaging of the olfactory pathway

**Fig. 1:**
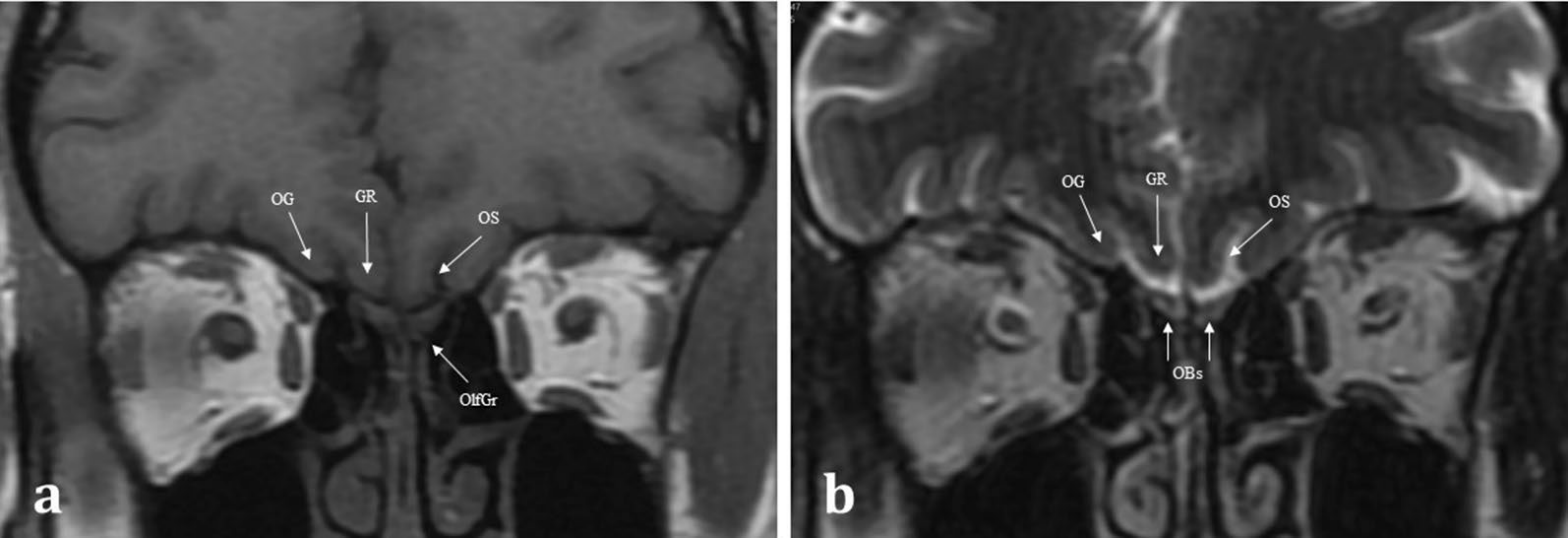
Normal Anatomy. Coronal T1WI (**a**) and T2WI (**b**) MRI images of the normal location and appearances of the olfactory bulbs (OBs). These lie within the olfactory grooves (OlfGr) of the anterior cranial fossa. Notice how the olfactory bulbs are surrounded by T2 hyperintense CSF. The olfactory tracts (not shown) are a posterior continuation of the olfactory bulbs and run within the olfactory sulci (OS). The olfactory sulcus is lateral to the gyrus rectus (GR) and medial to the orbital gyrus (OG)

**Fig. 2:**
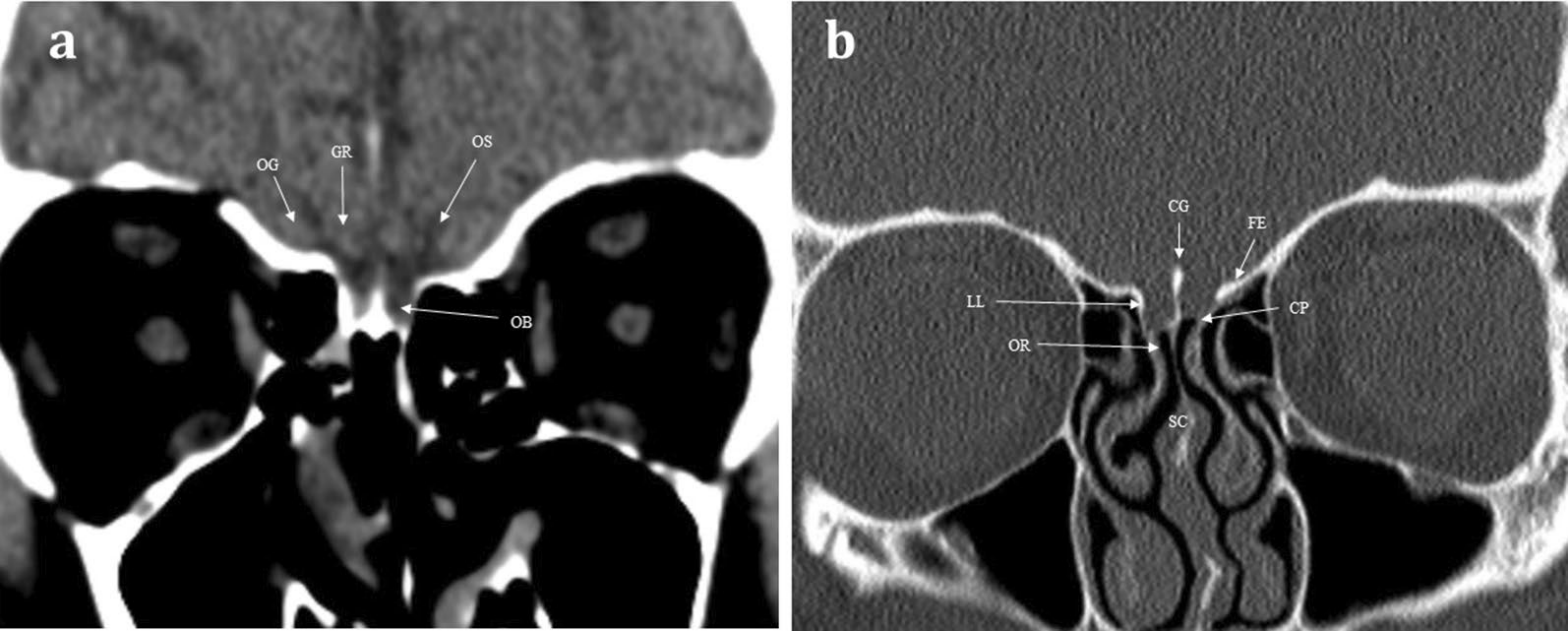
Normal anatomy. Coronal soft tissue (**a**) and bone window (**b**) CT images at the level of the olfactory bulb, demonstrating the normal anatomy of the sinonasal compartment (SC) and anterior skull base. The olfactory nerves within the SC and olfactory recess (OR) ascend intracranially via perforations within the cribriform plate (CP). The CP is bounded laterally via the lateral lamella (LL), the crista galli (CG) is seen as a midline projection, and the fovea ethmoidalis (FE) forms the roof of the ethmoid. The position of the olfactory bulb (OB) within the olfactory groove is just discernible on the soft tissue window image (**a**). The gyrus rectus (GR) and orbital gyrus (OG) are separated by the olfactory sulcus (OS)

Aromatic molecules within the air are inspired and directed to the posterosuperior region of each nasal cavity, to the olfactory neuroepithelium [4]. The olfactory neuroepithelium is made up of approximately 6–10 million neurons, occupying an area of 2.5cm^2^ [5]. The neurons combine to form nerve fascicles called fila olfactoria, which together form the first-order olfactory nerves. These first-order olfactory nerves serve as the interface between the peripheral and central olfactory systems. They ascend intracranially across the anterior cranial fossa via multiple perforations in the cribriform plate [4] (Figs. 1, 2). The fila olfactoria are normally difficult to discern on routine 1.5 T imaging, but are identifiable on higher magnetic field 3 T MRI [6].

**Fig. 3:**
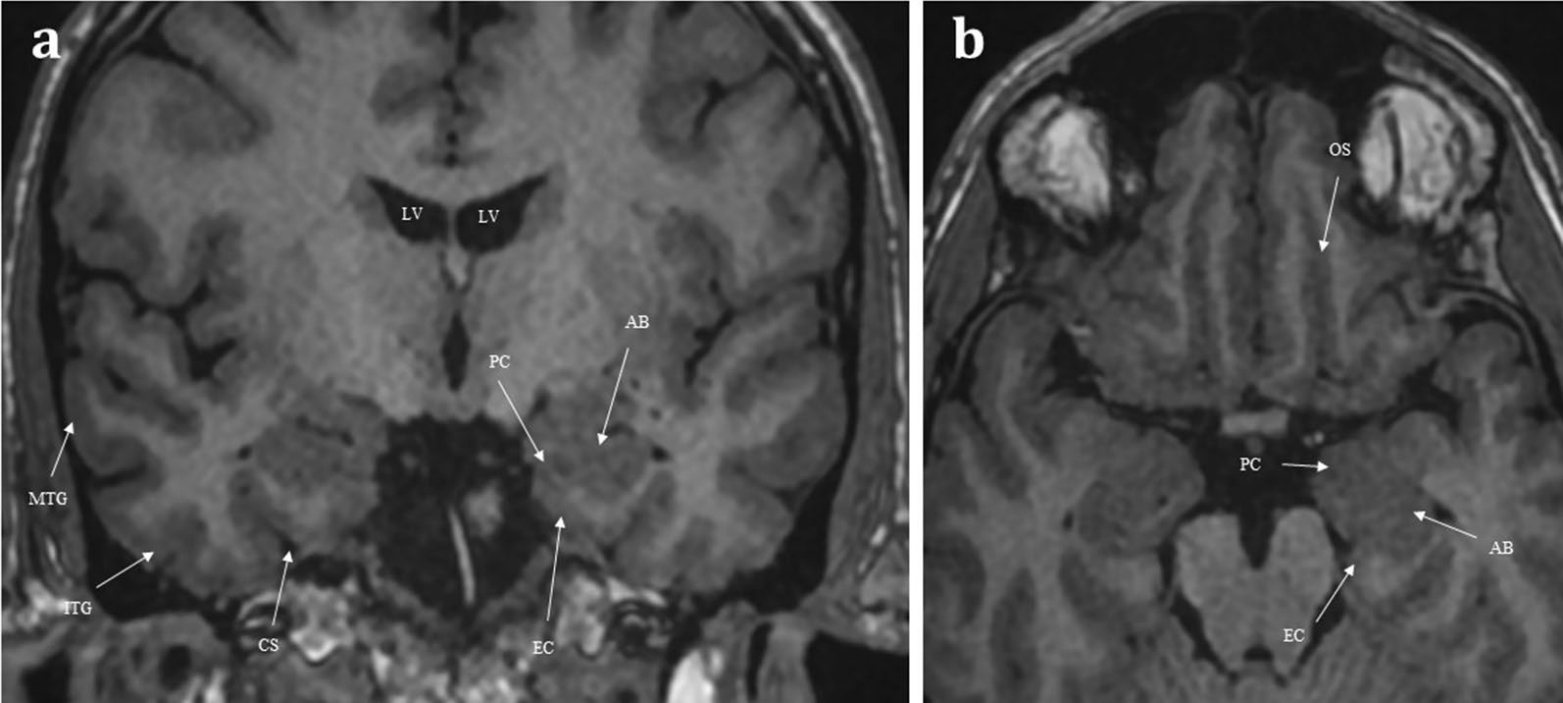
Coronal T1 weighted image taken at the level of the lateral ventricles (LV) and foramina of Monroe (**a**), and axial T1 weighted image of the inferior frontal lobe and mesial temporal lobes (**b**). The olfactory tracts (not pictured) run within the olfactory sulci (OS) and transmit second-order neurones to the central olfactory regions. These are housed within the mesial temporal lobe and include the parahippocampus, the entorhinal cortex (EC), the piriform cortex (PC), and the amygdaloid body (AB). Note the other sulcal landmarks including the collateral sulcus (CS) which bounds the entorhinal cortex inferiorly, as well as the inferior temporal gyrus (ITG), and the middle temporal gyrus (MTG)

The first-order olfactory nerve neurons synapse with second-order neurons within the ventral surface of the olfactory bulb, at the base of the frontal lobes. The two olfactory bulbs are located within the olfactory grooves and are surrounded by cerebral spinal fluid (CSF) [7]. They run a parallel course projecting posteriorly (Fig. 3). The olfactory tracts are a continuation of the olfactory bulbs, transmitting the second-order neurons. The olfactory tracts lie within the olfactory sulcus between the gyrus rectus medially and the orbital gyrus laterally (Fig. 1) [4]. The olfactory bulb measures 11–15 mm in length and 4–5 mm in width. The olfactory tract is longer, measuring up to 30 mm and tapering in width from 5 to 2 mm as it passes posteriorly [7]. Olfactory bulb volume correlates with olfactory function [8]. Experimental in vitro cadaveric imaging of the olfactory bulb using 9.4 T MR-microscopy has managed to identify six separate signal intensity layers representing the distinct cellular lamination pattern of the olfactory bulb [9].

The olfactory tracts containing the second-order neurons pass superolaterally over the optic nerves and suprasellar cistern, terminating at the olfactory trigone which lies just above the anterior clinoid process [4]. These second-order neurones then enter the olfactory striae (medial, lateral and intermediate) and then project into the mesial temporal lobe. The mesial temporal lobe houses the central olfactory regions including the entorhinal cortex, piriform cortex, amygdala, and the parahippocampus (Fig. 3) [7]. There are myriad interconnections between these central olfactory regions and the rest of the brain parenchyma, including the mediodorsal thalamus, orbitofrontal cortex, temporal cortex and other regions of the limbic system. This vast network of intracerebral connections enables one’s sense of smell to be intimately integrated with taste, memories, and emotions [4].

## Imaging

CT and MRI provide complimentary information in imaging the olfactory pathway [6]. CT is typically the initial imaging modality for suspected “conductive” olfactory problems, in order to identify sinonasal pathology that limits the interaction between aromatic molecules and the olfactory epithelium [10]. At our institution, axial non-contrast CT is performed through the sinonasal compartment and anterior skull base at 0.75–1-mm slice thickness to enable multiplanar reformats in both the sagittal and coronal planes. CT provides the exquisite bony detail required for assessment of the integrity of the ethmoid roof and anterior skull base and enables the exclusion of intrinsic skull pathologies or secondary skull involvement due to other disease processes (Fig. 2). This includes evaluation of bone remodelling and/or destruction associated with inflammatory processes or tumours [11]. Conventional CT imaging, as opposed to cone beam CT imaging, also provides soft tissue information that allows assessment of the sinonasal contents, extracranial soft tissues, and intracranial contents.

MR imaging enables more detailed soft tissue assessment of both the peripheral and central components of the olfactory pathways [6, 12]. At our institution, the MR imaging protocol for olfactory dysfunction includes coronal T2 and T1 weighted imaging with a slice thickness of 2 mm covering the sinonasal compartment, anterior skull base, and anterior cranial fossa contents (Fig. 1). The coronal T2 imaging enables assessment of volume and signal change within the olfactory bulbs and tracts. If there is concern regarding a potential CSF leak, an additional gradient echo CISS (constructive interference in steady state) sequence to cover the anterior skull base is performed, with volumetric 1-mm slices acquired to enable multiplanar reformation. Additional contrast enhanced and diffusion weighted imaging may be necessary to aid differentiation between benign and malignant sinonasal pathologies [10]. The routine imaging protocol includes axial T2 brain imaging to assess the limbic and mesial temporal lobe structures as well as volumetric and coronal T2 and/or FLAIR imaging of the temporal lobes as per standard epilepsy protocols. If there is a prior history of trauma, gradient recall echo- or preferably susceptibility weighted imaging- is also acquired [6].

## Peripheral sinonasal compartment pathology

Pathology within the nasal cavity can obstruct the passage of air to the receptors of the olfactory epithelium and can also cause inflammation of the olfactory epithelium. Both of these processes can lead to olfactory disturbance [6, 13]

**Fig. 4:**
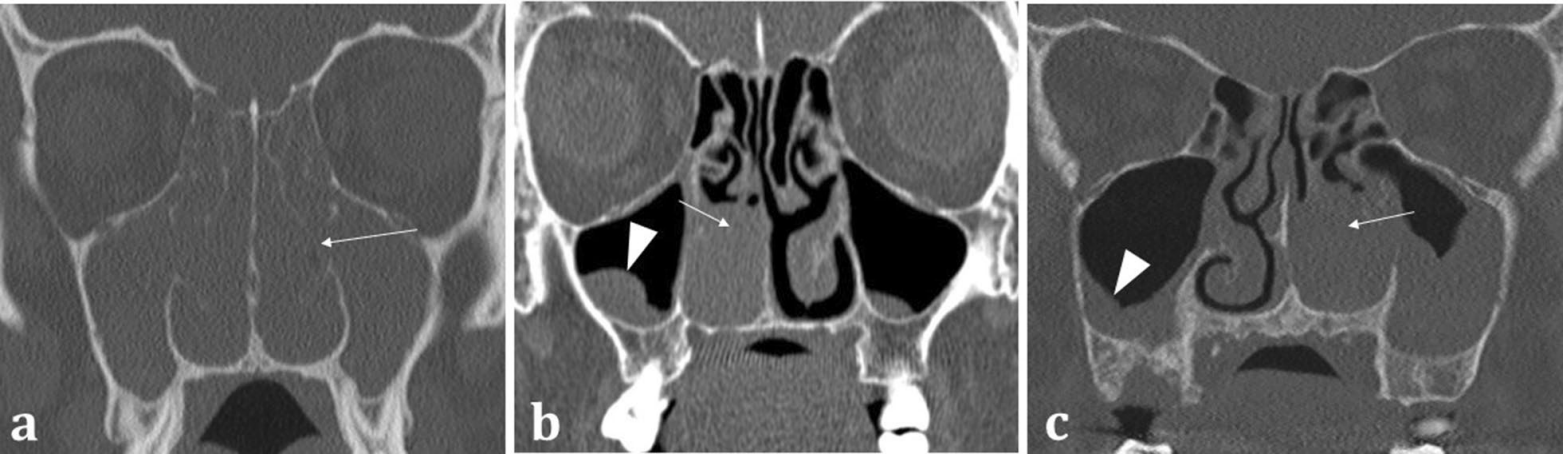
Coronal bone window CT images of three different patients with sinonasal disease: 51-year-old female with 4-year history of nasal congestive symptoms and anosmia diagnosed with sinusitis (**a**); 70-year-old female patient presenting with bloody nasal discharge and right nasal blockage diagnosed with sinonasal carcinoma (**b**); and 70-year-old female presenting with nasal congestion diagnosed with malignant melanoma (**c**). Pansinusitis with complete opacification of the paranasal sinuses (**a**). The bilateral nature of the disease is a sign of benignity whilst the rarefaction and thinning of the bony architecture (arrowed) suggests a more chronic process. Conversely unilateral opacification as demonstrated in (**b**) and (**c**) is suspicious for a malignant process. Notice the bony destruction of the inferior turbinate in both cases (arrowed in **b** and **c**), which can be difficult to discern from rarefaction (arrowed, **a**); however, this must be assessed in the context of unilateral disease. The imaging appearances in (**b**) and (**c**) are strikingly similar on both CT and MRI (not shown) and were only distinguishable on histology, which confirmed sinonasal squamous cell carcinoma (**b**) and malignant melanoma (**c**). Incidental note is made of bilateral maxillary sinus mucus retention cysts (**b**), identifiable by their rounded, convex contour, and fluid density (arrowhead, **b**), in contrast to the concave maxillary mucosal thickening (arrowhead, **c**)

The sinonasal compartment should normally be air-filled; opacification of the sinonasal compartments on CT suggests the presence of underlying disease processes [14]. In the setting of abnormal sinonasal opacification, evaluation of the surrounding bone architecture is essential to identify any suspicious bony destruction that may portend an aggressive and potentially malignant process [12]. Bony expansion with thinning (termed “rarefaction”), or remodelling with thickening of the bone, both suggests a slow, progressive, and usually benign process (Fig. 4a). The density of the opacification on CT can also help discriminate between various pathologies. Hyperdense opacification suggests benign disease such as inspissated secretions in sinusitis, fungal elements in non-invasive allergic fungal sinusitis, or blood in the context of trauma. The exception is hypervascular tumours such as melanoma, as these can bleed [12]. Hypodensity on CT represents either secretions or malignancy, which can be distinguished on MRI [15].

**Fig. 5:**
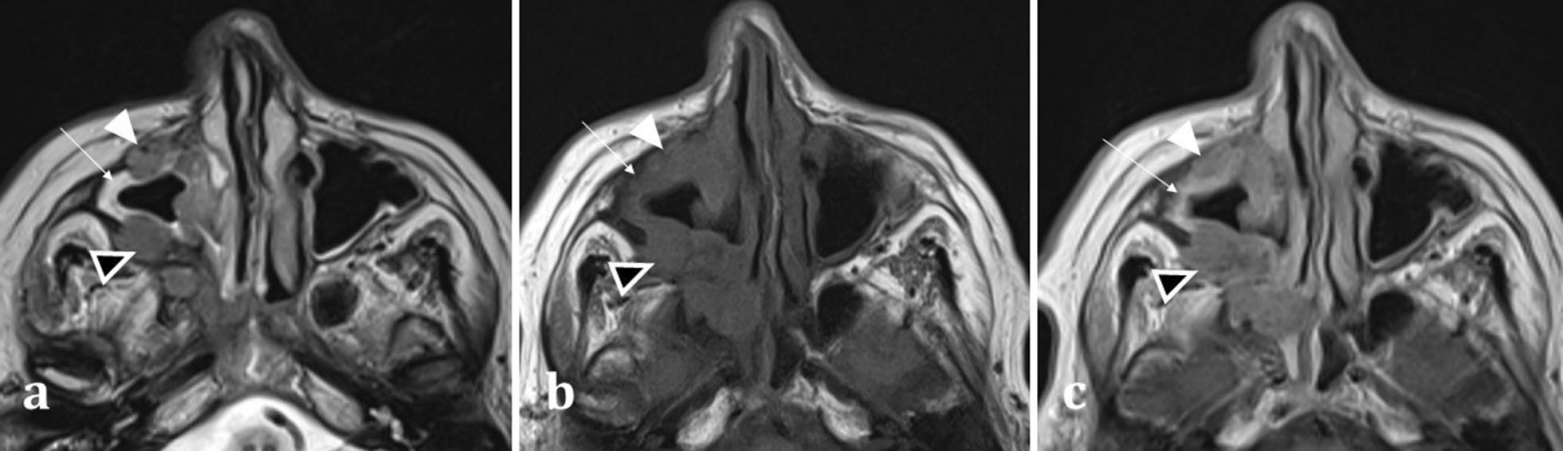
Axial MRI images of a 66-year-old female patient with right maxillary sinus squamous cell carcinoma (SCC). She presented with symptoms of chronic rhinosinusitis for which a CT sinuses was performed (not shown) demonstrating unilateral maxillary sinus disease, suspicious for malignancy. Axial T2 weighted image (**a**) exhibits an isointense soft tissue lesion (white arrowhead) permeating and extending through the right maxillary sinus, which can be differentiated from the high T2 signal normal mucosa (arrow). Both the SCC (white arrowhead) and normal mucosa (arrow) are hypointense on T1W imaging (**b**). On T1W post-contrast imaging (**c**), the lesion demonstrates less enhancement (white arrowhead) compared to the adjacent hyperenhancing sinonasal mucosa (arrow) (**c**). Transpatial spread into the right pterygopalatine fossa is also identifiable on all sequences (black arrowheads with white outline)

MRI assists in differentiation between solid soft tissue elements of a tumour and the fluid characteristics of secretions. Secretions are T2 hyperintense and T1 hypointense, whereas primary sinonasal neoplasms are usually T2 intermediate- or less commonly T2 hyperintense- and T1 hypointense [15] (Fig. 5). Post-contrast MR imaging aids in delineation of sinonasal soft tissue masses. Solid components of sinonasal tumours enhance, whilst the haemorrhagic, necrotic, or cystic components do not. Sinonasal tumours can be differentiated from thickened mucosa (peripheral enhancement) and secretions (which do not enhance) based on their enhancement patterns (Fig. 5) [14].

**Fig. 6:**
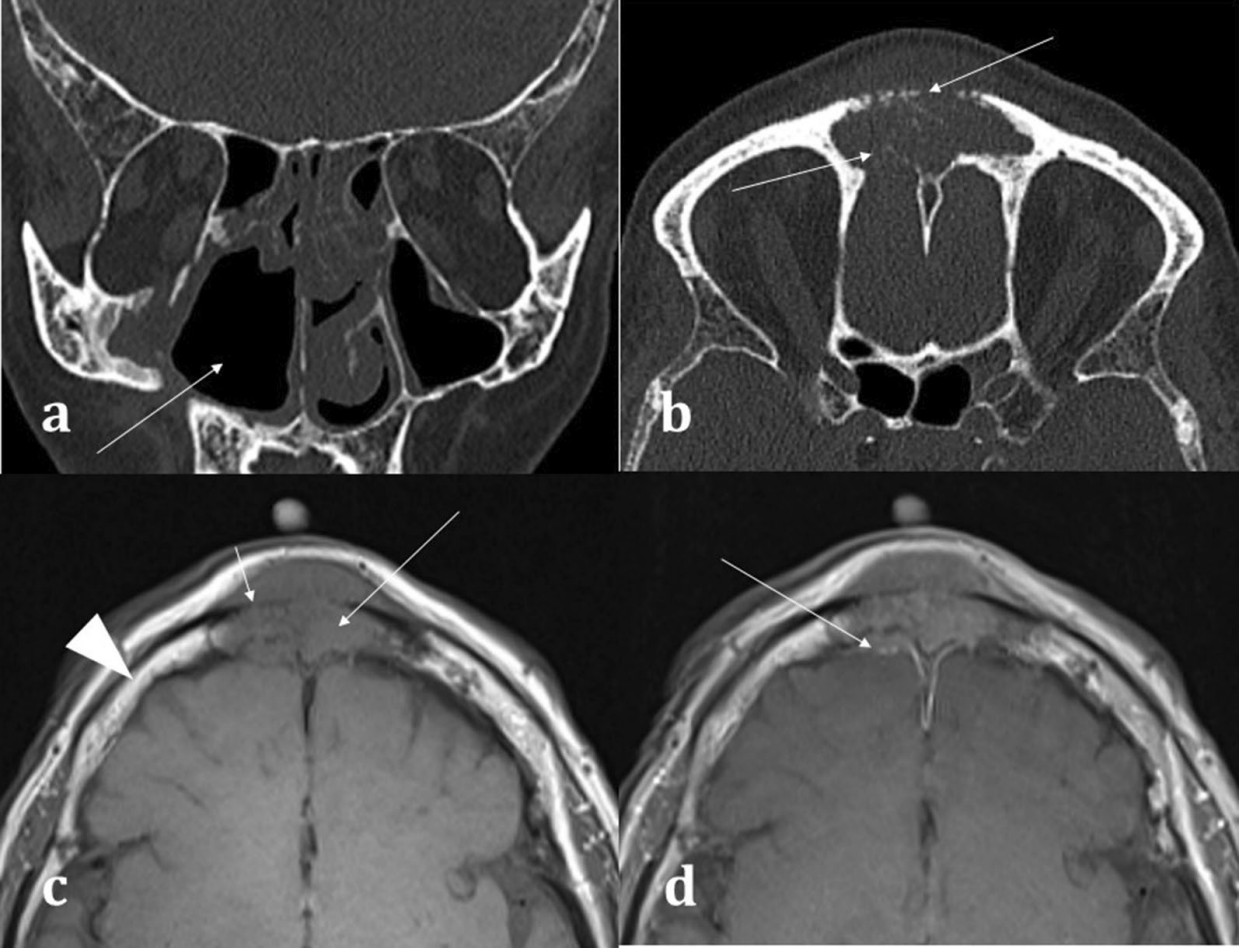
Transpatial spread of recurrent of sinonasal squamous cell carcinoma (SCC), in the same patient as shown in Fig. 4b. Coronal bone window CT **a** demonstrates the right maxillectomy. Axial bone window CT **b** shows a permeative process within the frontal sinus causing cortical destruction (arrowed). T1WI demonstrates recurrent SCC involving the frontal sinus (**c**), expanding the sinus and infiltrating the bone (long arrow) with loss of the normal fatty marrow signal (arrowhead for comparison). There is clear evidence of inner and outer table cortical breach on T1WI with loss of the normal hypointense cortical rim (short arrow) placing the patient at risk of intracranial disease. The lesion demonstrates mild enhancement (**d**), with associated dural thickening and enhancement (arrow) indicating transpatial spread

MRI is best suited for demonstrating any transpatial spread of sinonasal disease, including intracranial spread [12]. This includes perineural spread of disease, seen particularly in adenoid cystic carcinoma and squamous cell carcinoma [12]. This is demonstrated on MRI as nerve thickening and/or enhancement, and loss of perineural fat [16]. Widening of the neural foramina may also be seen, especially foramina relating to branches of the trigeminal nerve [17]. Orbital and anterior skull base invasion in both malignant and infective conditions are also best seen on MRI [16]. On coronal imaging, loss of the normal low signal anterior skull base cortical margin, loss of the normal T1 hyperintense marrow signal, and frank breach of the anterior cranial fossa on post-contrast T1WI (Fig. 6) are sensitive indicators of bone infiltration and transcalvarial spread of disease [15].

### Benign inflammatory diseases

**Fig. 7:**
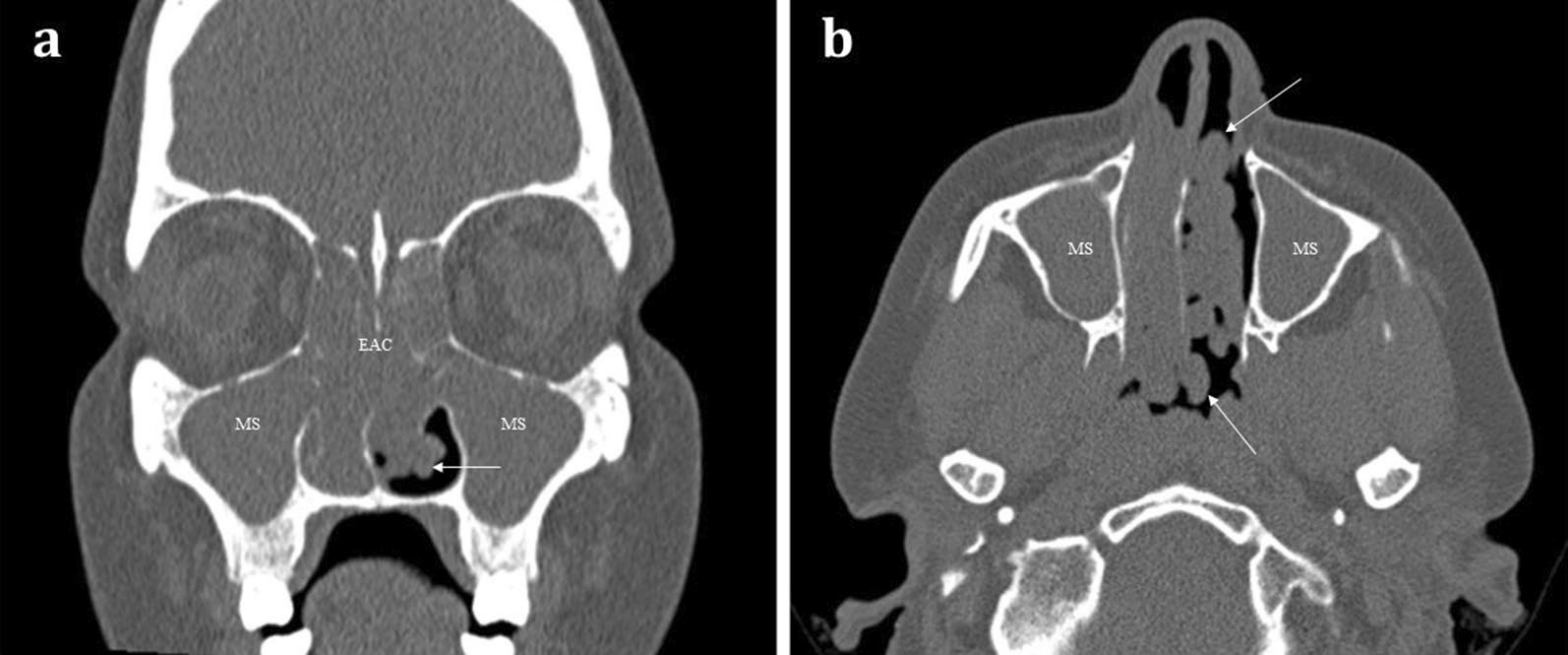
48-year-old female with long standing nasal congestion and rhinorrhoea. Coronal (**a**) and axial (**b**) bone window CT images demonstrating extensive sinonasal polyposis with extension into the nasopharynx. Bilateral involvement is suggestive of benign disease. Note the characteristic rounded contours (arrows) which are very suggestive of polyposis. There is also complete opacification of the maxillary sinuses (MS) and ethmoid air cells (EAC)

*Sinonasal polyposis* is a benign inflammatory mucosal condition that most commonly affects the maxillary sinuses and often extends into the middle meatus of the nasal cavity (Fig. 7). It is characterised by its rounded contours. The presence of more extensive sinonasal polyposis, particularly in the upper meatus and the posterior portion of the middle meatus, can significantly impede olfactory function [18].

*Chronic rhinosinusitis* is common, and the severity of chronic sinusitis is correlated with reduced olfactory bulb volumes [13]. Sinonasal polyposis often coexists with chronic rhinosinusitis. The role of CT is to assess the pattern of sinonasal drainage impairment, to guide potential endoscopic approach [16]. Prompt diagnosis allows for early treatment which can increase olfactory bulb volumes and improve the patient’s sense of smell [19]. Mucosal thickening and sinus opacification can occur in both acute and chronic sinusitis. The presence of an air-fluid level distinguishes the acute form from the chronic form [15, 16].

*Fungal sinusitis* can be either non-invasive (subcategorised as allergic or fungal ball/mycetoma forming) or invasive (subcategorised as acute invasive, chronic invasive, or the very infrequently occurring chronic granulomatous) [20]. Allergic non-invasive fungal sinusitis is most common. Although olfactory dysfunction is not considered part of the diagnostic criteria, patients can experience significant olfactory disturbance [21]. It is characterised by the presence of hyperdense serpiginous fungal elements (which can even be calcified) completely opacifying and expanding/remodelling the sinus [21].

**Fig. 8:**
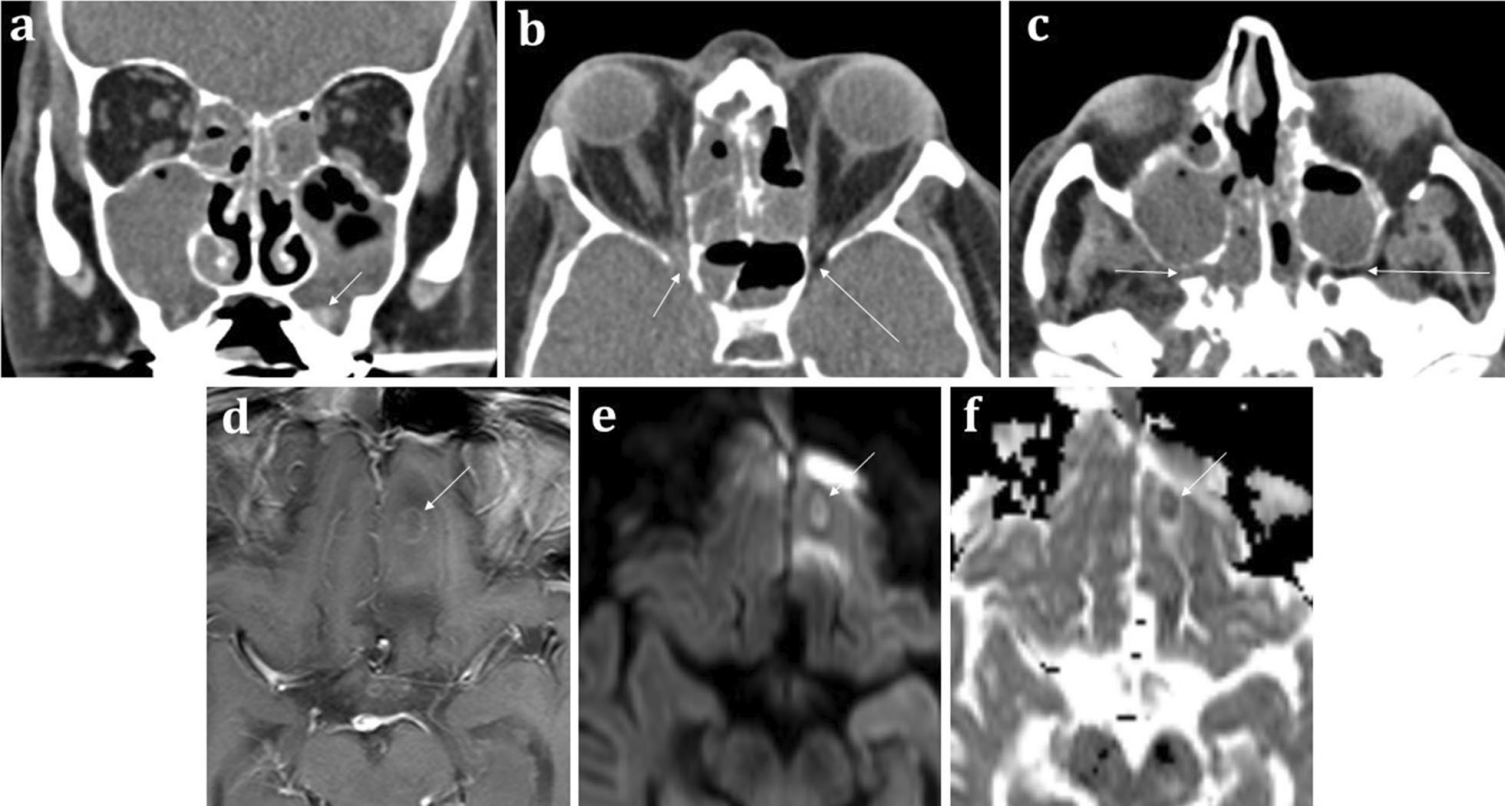
60-year-old male with neutropenic sepsis on a background of allograft bone marrow transplant for acute myeloid leukaemia. Coronal soft tissue CT **a** demonstrates “bubbly” aerated secretions within the maxillary sinuses and ethmoid air cells bilaterally suggestive of acute sinusitis. Hyperdense regions are indicative of inspissated secretions or fungal elements (arrowed). Axial soft tissue CT images **b**, **c** illustrate subtle abnormal soft tissue at the right orbital apex (short arrow, **b**) and the right pterygopalatine fossa (short arrow, **c**), which suggests trans-spatial spread of disease. Appearances are consistent with acute invasive fungal sinusitis. On the contralateral side, the normal fat within these spaces is preserved (long arrows in **b** and **c**). Axial T1W post-contrast (**d**), DWI (**e**), and ADC map (**f**) MRI sequences demonstrate a left inferior frontal lobe ring enhancing lesion with central diffusion restriction consistent with an intracranial abscess (arrowed)

*Acute invasive fungal sinusitis* conversely is potentially life-threatening and occurs in immunocompromised patients. It has a propensity for the ethmoid air cells and sphenoid sinuses [20]. Invasion and obliteration of the periantral, pterygopalatine, or orbital apex fat (Fig. 8) portend life-threatening intracranial spread [20]. Invasive features, however, may not always be present on CT and severe mucosal thickening- though non-specific- is the most sensitive finding [20].

**Fig. 9:**
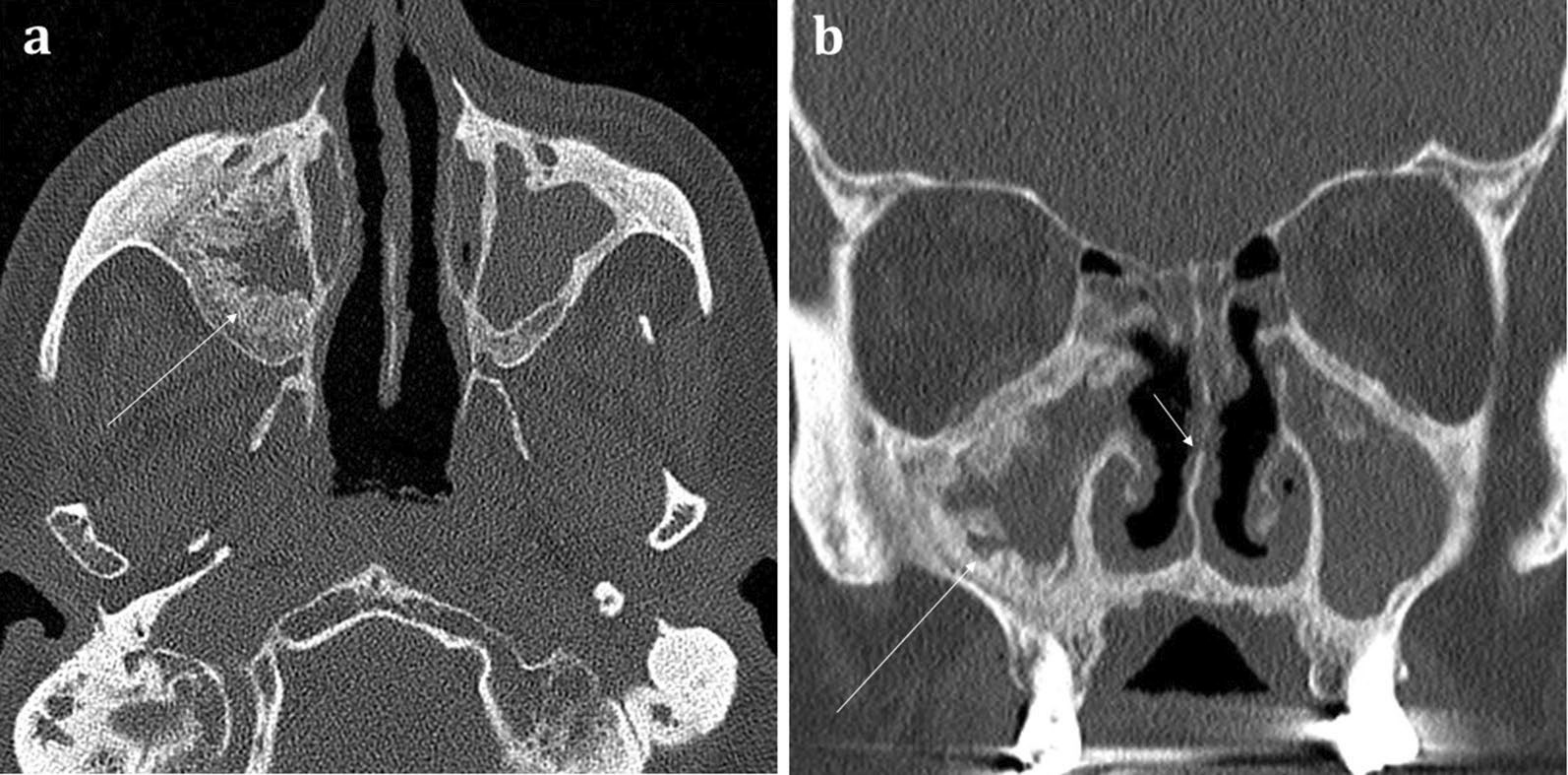
Axial (**a**) and Coronal (**b**) bone window CT images of a 71-year-old patient with known granulomatosis with polyangiitis (GPA) suffering from chronic nasal obstructive symptoms and nasal crusting. There is widespread mucosal thickening throughout the sinuses with evidence of “frond-like” chronic remodelling and osteitis (long arrows). On the coronal image (**b**), erosion of the middle turbinates and irregularity of the nasal septum can be seen (short arrow)

*Granulomatosis with polyangiitis* (GPA) can cause rhinosinusitis with nasal obstruction. As the disease becomes established, sinonasal erosion with osteitis and osteogenesis is common. There is a predilection for nasal septum involvement and perforation (Fig. 9) [11, 16]. Of note, T-cell variant of sinonasal lymphoma can be indistinguishable from GPA-associated rhinosinusitis [22]. Whilst cocaine-associated sinonasal disease can appear similar to GPA, unlike GPA it has a propensity to also cause soft and hard palate destruction [16].

### Malignant sinonasal disease

*Sinonasal cancers* are uncommon and account for 3% of head and neck cancers. Head and neck cancers themselves represent just 1% of all cancers [23]. The clinical presentation is non-specific, and there is considerable overlap with inflammatory sinus disease, which can coexist. Anosmia is a late symptom, along with facial swelling, visual disturbance, and cranial nerve palsies [12]. Unfortunately, patients often present at an advanced stage of disease with invasion into surrounding structures [14].

*Squamous cell carcinoma (SCC)* is responsible for over 50% of sinonasal cancers [23] and is characterised by aggressive bone destruction, with necrotic soft tissue on CT. It typically affects the maxillary sinus unilaterally, or less commonly the nasal cavity where it may present clinically with a non-healing ulcer [24]. SCC has non-specific MRI characteristics exhibiting T1 isointensity, T2 intermediate, or slight T2 hyperintensity (Fig. 5), with moderate enhancement [12]. Human papillomavirus (HPV)-related SCC usually affects the nasal cavity and has an improved prognosis compared to non HPV-related SCC [25]. Synchronous nasopharyngeal SCC is possible, and the aerodigestive tract should routinely be reviewed [26].

*Non-SCC sinonasal malignancies* are much less common and include other epithelial cancers such as adenocarcinoma, minor salivary gland tumours, and non-epithelial tumours such as lymphoma and melanoma [23]. As with SCC, anosmia and olfactory dysfunction are typically late manifestations. Their imaging appearances can be indistinguishable from the more common SCC and may only be differentiated on histology (Fig. 4) [12].

## Central sensorineural pathology

### Congenital abnormalities

Unlike acquired causes of anosmia (detailed below), congenital anosmia is rare. A diagnosis of congenital anosmia can be made in patients with olfactory dysfunction confirmed on functional testing and with no recollection of ever having had smell sensation [6]. Congenital anosmia can be seen as part of a syndrome, or in the context of isolated congenital anosmia in which anosmia is the sole complaint [27].

**Fig. 10:**
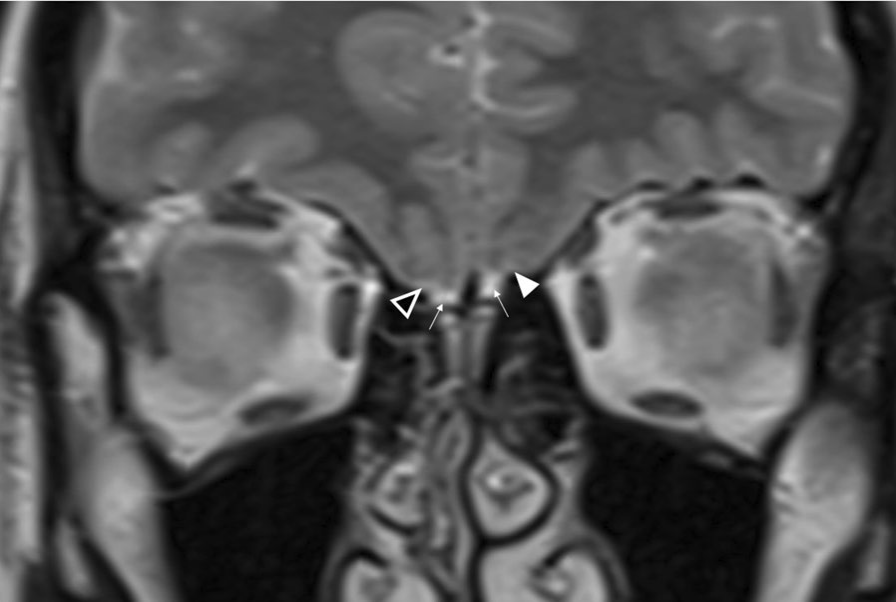
18-year-old female presenting with no memory of ever having had a sense of smell. Coronal T2 weighted image demonstrates absence of the olfactory bulbs and olfactory tracts bilaterally (arrowed). There is additional blunting of the right olfactory sulcus (black arrowhead with white outline) whilst the left olfactory sulcus is preserved (arrowhead). The history and imaging findings are consistent with congenital anosmia

**Fig. 11:**
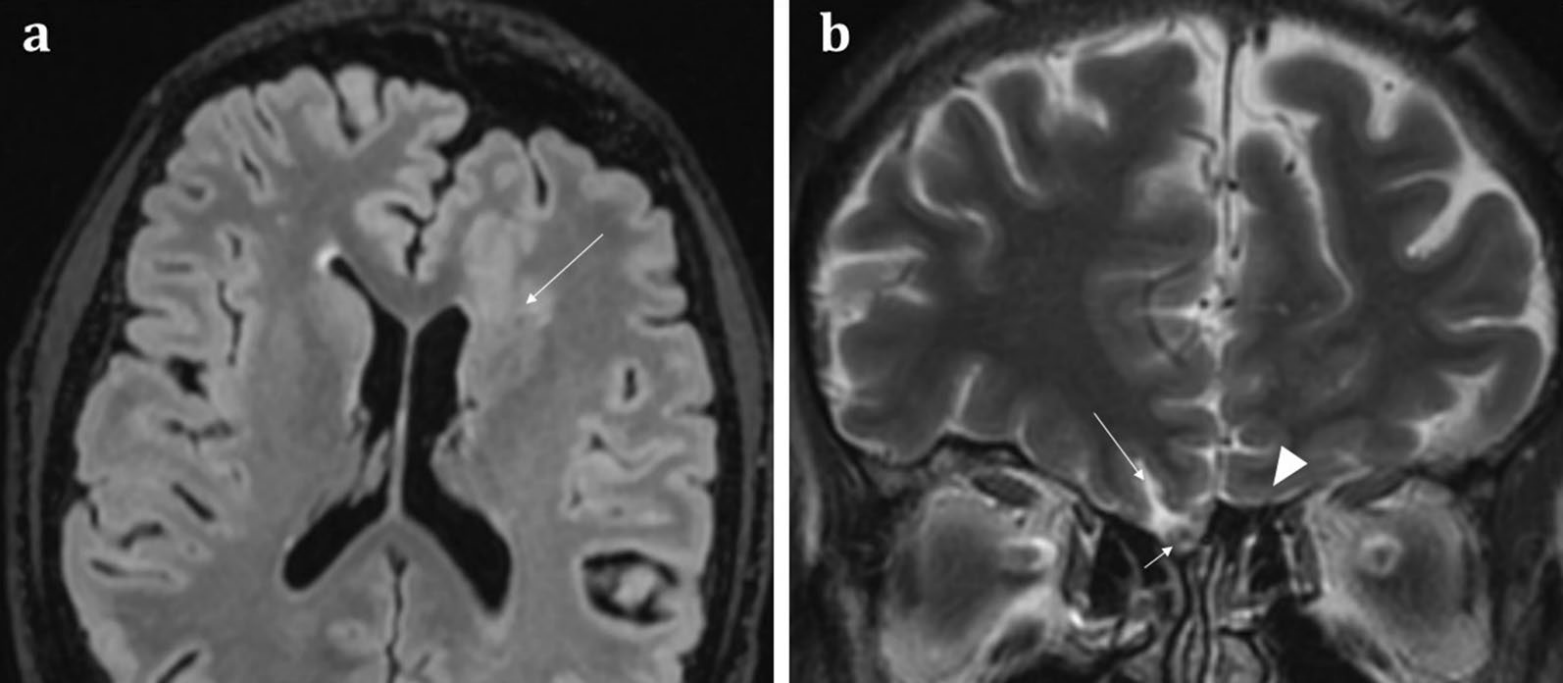
51-year-old male with a history of known focal epilepsy secondary to grey matter heterotopia. Axial FLAIR sequence (**a**) demonstrates unilateral hypoplastic left cerebral hemisphere with focal subependymal grey matter heterotopia within the left frontal region (arrowed). Coronal T2WI (**b**) also demonstrates an associated ipsilateral complete absence of both the olfactory bulb and olfactory sulcus (arrowhead). In comparison, the right olfactory bulb (short arrow, **b**) and olfactory sulcus (long arrow, **b**) are preserved

MR imaging plays an important role in the diagnosis of congenital anosmia, not only for characterising any related syndromic anatomical deficits, but also in assessing the olfactory apparatus, or lack thereof. Congenital olfactory problems are characterised by any combination of: (1) olfactory tract and/or olfactory bulb volume reduction, or even complete absence (Fig. 10), and (2) normal, hypoplastic, or aplastic “flattening” of the olfactory sulci unilaterally or bilaterally (Fig. 11) [28]. Coronal T2 fat-saturated MRI sequences provide optimal imaging for identification of the olfactory sulci and for evaluation of the olfactory bulbs and tracts along their entire lengths (for comparison to normal MRI anatomy see Fig. 1).

Congenital anosmia can be associated disorders of the paramedian structures (including corpus callosal dysgenesis, hypothalamic and pituitary problems, lobar holoprosencephaly, and septo-optic dysplasia), as well as migrational abnormalities (polymicrogyria, pachygyria, and grey matter heterotopia) (Fig. 1) [29]. CHARGE syndrome (Coloboma, Heart defects, Atresia of the choanae, Retardation of growth, Genital and/or urinary abnormalities, Ear anomalies and/or deafness) is associated with both congenital anosmia, as well as olfactory problems arising from choanal atresia [29].

*Kallmann syndrome* specifically describes congenital anosmia with hypogonadotrophic hypogonadism and presents with delayed onset or absent puberty where anosmia can be an incidental finding in the clinical history. Kallmann syndrome results from a variety of genetic mutations, the most common of which demonstrates an X-linked pattern of inheritance [29].

*Isolated congenital anosmia* without endocrine disorder is more common compared to Kallmann syndrome [27, 30]. However, it is often not possible to discriminate true congenital anosmia from childhood acquired anosmia secondary to early viral infection or trauma [31]. Nonetheless, a depth of the olfactory sulcus of ≤ 8 mm has been suggested as specific for confirming isolated congenital anosmia [30].

### Toxins and infection

Exposure to both viral infections and toxins has been associated with impairment of smell. Toxins account for an estimated 1–5% of all olfactory disorders and may be transient or permanent [32]. Postviral anosmia has recently become a topic of increased interest due to the COVID-19 pandemic, with anosmia having been reported as a common symptom of the infection [33]. Spontaneous recovery from postviral anosmia is seen in one-third of cases and can take up to 2 years [6]. It has been demonstrated that patients with postviral olfactory dysfunction have reduced olfactory bulb volumes on MRI compared to control groups [34]. Moreover, initial smaller olfactory bulb volumes at diagnosis may represent a poor prognostic marker for recovery of sense of smell – total olfactory bulb volumes of less than 40cm^3^ suggest a negligible chance of recovery [35].

### Olfactory neuroblastoma

**Fig. 12:**
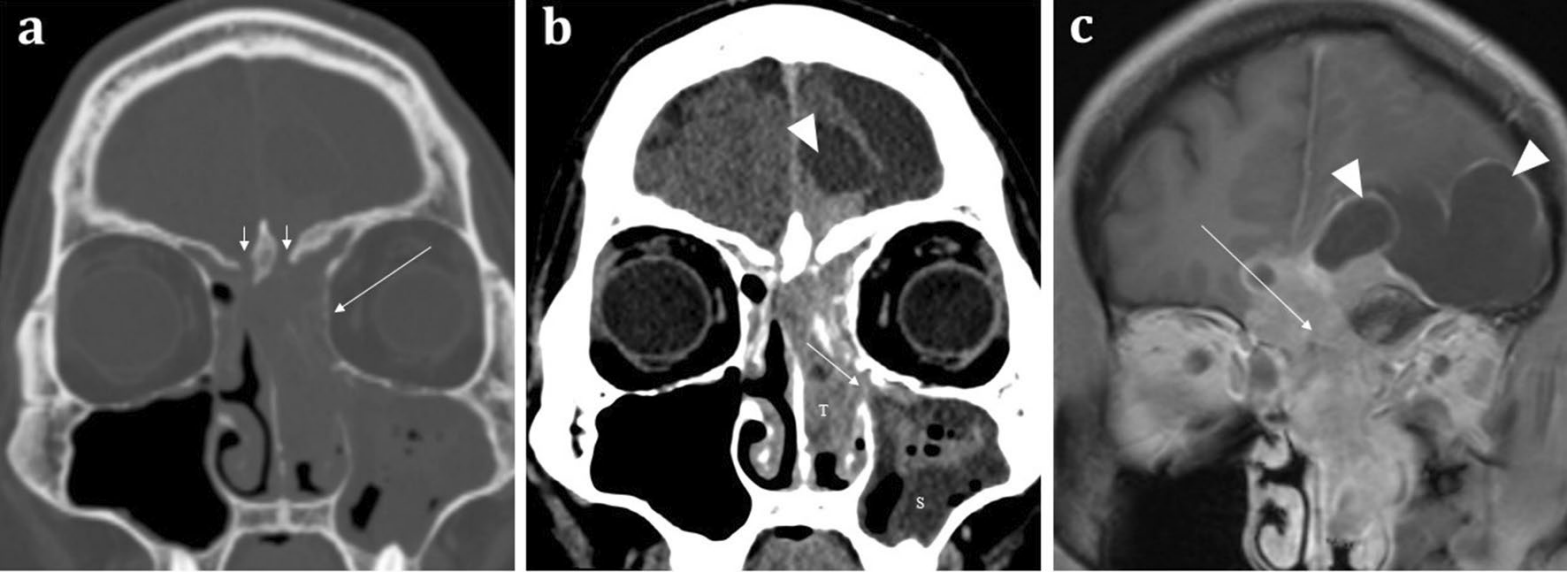
Olfactory neuroblastoma (Esthesioneuroblastoma) in a 41-year-old male presenting with a 2-month history of change in behaviour and anosmia. Coronal bone (**a**) and soft tissue window (**b**) CT images demonstrate a large anterior skull base mass centred upon the olfactory neuroepithelium. There is extensive bone destruction of the left and right cribriform plates and turbinates (short arrows, **a**), and thinning of the medial wall of the left orbit (long arrow, **a**). There is also inferior extension of the tumour (T) through the ethmoid air cells into the nasal cavity and extending through the left middle meatus into the maxillary sinus (arrowed, **b**). The soft tissue density of the tumour (T) is distinct from the left maxillary sinus fluid contents (S). Note the fluid density intracranial “capping cysts” at the left frontal lobe (arrowhead, **b**). Coronal T1WI with contrast (**c**) depicts a tumour with moderately intense contrast uptake, conforming to a “dumbbell-shape” with a “waist” at the cribriform plate (arrowed, **c**). Again, note the presence of T1 hypointense “capping cysts” at the intracranial margin, which are a distinguishing feature of these tumours (arrowheads, **c**)

Primary tumours arising from the olfactory apparatus are rare. Olfactory neuroblastomas (or esthesioneuroblastomas) arise from the olfactory neuroepithelium and may lead to olfactory dysfunction [22]. However, patients more commonly present with nasal obstruction, epistaxis, and ocular disturbance. The tumour demonstrates a bimodal peak in the second and sixth decades [22]. Olfactory neuroblastomas are typically located at the olfactory neuroepithelium and are classically “dumbbell” shaped with the “waist” of the mass formed at the cribriform plate (Fig. 12) [22]. They demonstrate soft tissue density on CT and calcifications may be seen. On MRI, relative to grey matter, these tumours are isointense on T1, isointense-to-hyperintense on T2 weighted sequences, and enhance heterogeneously [22]. Olfactory neuroblastomas are slow-growing and often large at presentation with significant bony remodelling. However, destruction of the adjacent cribriform plate, fovea ethmoidalis, and lamina papyracea and extension into the anterior cranial fossa can also be present [11]. The intracranial extent is often better delineated on MRI than on CT. With intracranial extension, “capping cysts” at the margin between the tumour and the brain can often be identified (Fig. 10). The presence of intracranial “capping cysts” is highly suggestive of an olfactory neuroblastoma and is not seen in other anterior skull base lesions [36]. Lateral breach into the orbits, inferior extension into the nasal cavity, and posterior involvement of the sphenoid sinuses are all also frequently seen [22].

### Olfactory groove meningiomas

**Fig. 13:**
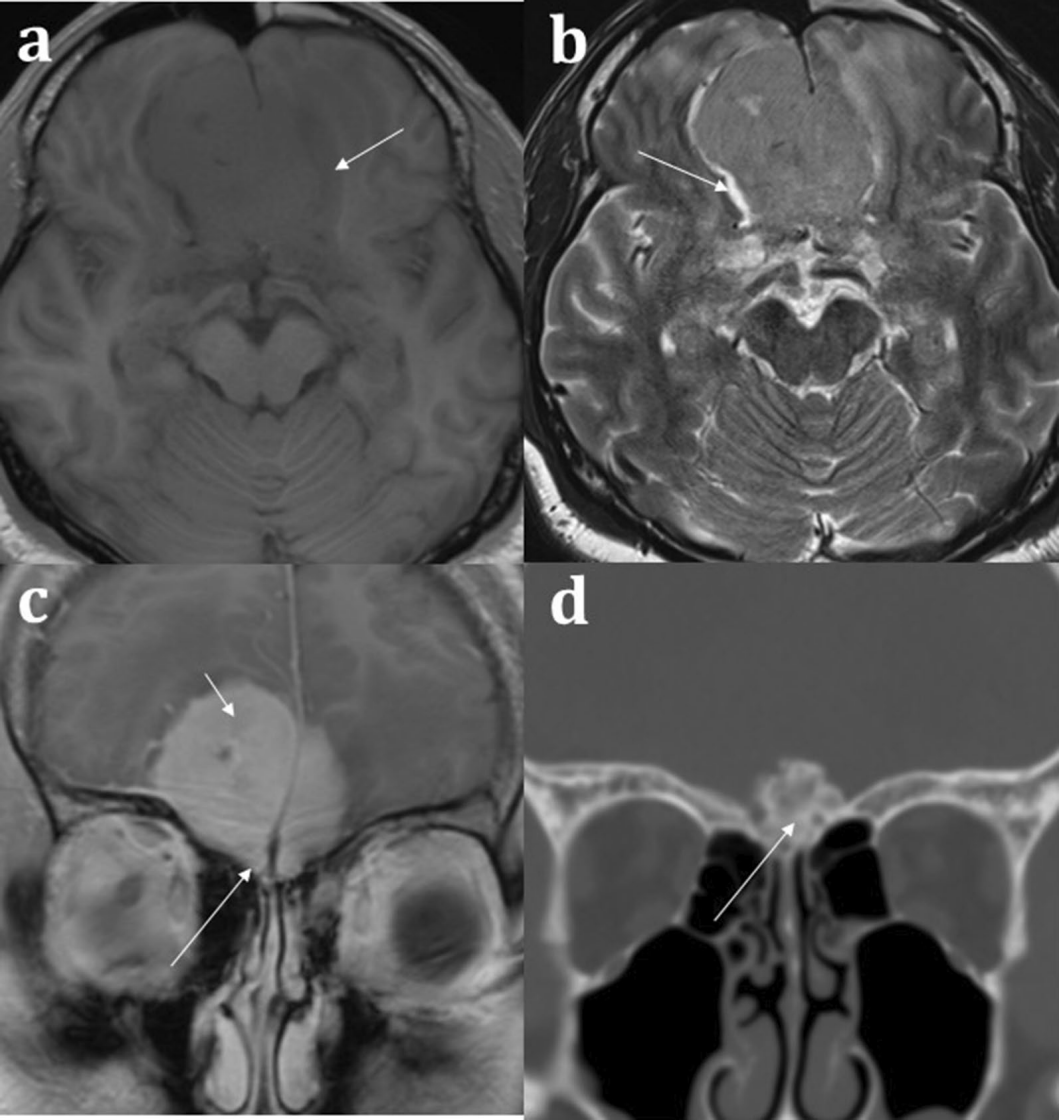
Olfactory groove meningioma. 51-year-old male presenting with headache. A large central intermediate intensity lesion is seen on T1W (**a**) and T2W imaging (**b**). It occupies the anterior skull base centrally crossing the midline and demonstrates grey matter immediately adjacent to the lesion (arrow, **a**) and a CSF cleft (arrow, **b**) indicative of an extra-axial location. The lesion demonstrates homogenous intense enhancement following contrast administration (short arrow, **c**). The coronal bone window CT (**d**) demonstrates hyperostosis of the underlying anterior cranial fossa (long arrows), typical for a meningioma. Despite the marked mass effect on the olfactory sulcus and intimate association with the olfactory groove (long arrow, **c**), the patient only complained of a mild olfactory disturbance

Olfactory groove meningiomas account for 8–13% of intracranial meningiomas [37]. They tend to present with headache, visual disturbance, or frontal symptoms including dementia-type symptoms [37]. Olfactory groove meningiomas exhibit imaging features typical for a meningioma, including a CSF cleft, hyperostosis of the underlying bone, isointensity to grey matter on T1WI, iso-to-hyperintensity on T2 weighted imaging, and homogenous post-contrast enhancement (Fig. 13) [6, 37]. A small case series concluded that despite their close relation to the olfactory apparatus, olfactory groove meningiomas can but do not consistently cause hyposmia/anosmia [38]. In addition, when present, ansomia may not resolve with successful surgical resection. Furthermore, olfactory disturbance ipsilateral to the tumour location is a common sequelae post-resection [37]. Olfactory groove meningiomas are a common cause of the rare Foster Kennedy syndrome (direct optic nerve compression causing unilateral optic atrophy with contralateral papilloedema from raised intracranial pressure and anosmia).

### Trauma

**Fig. 14:**
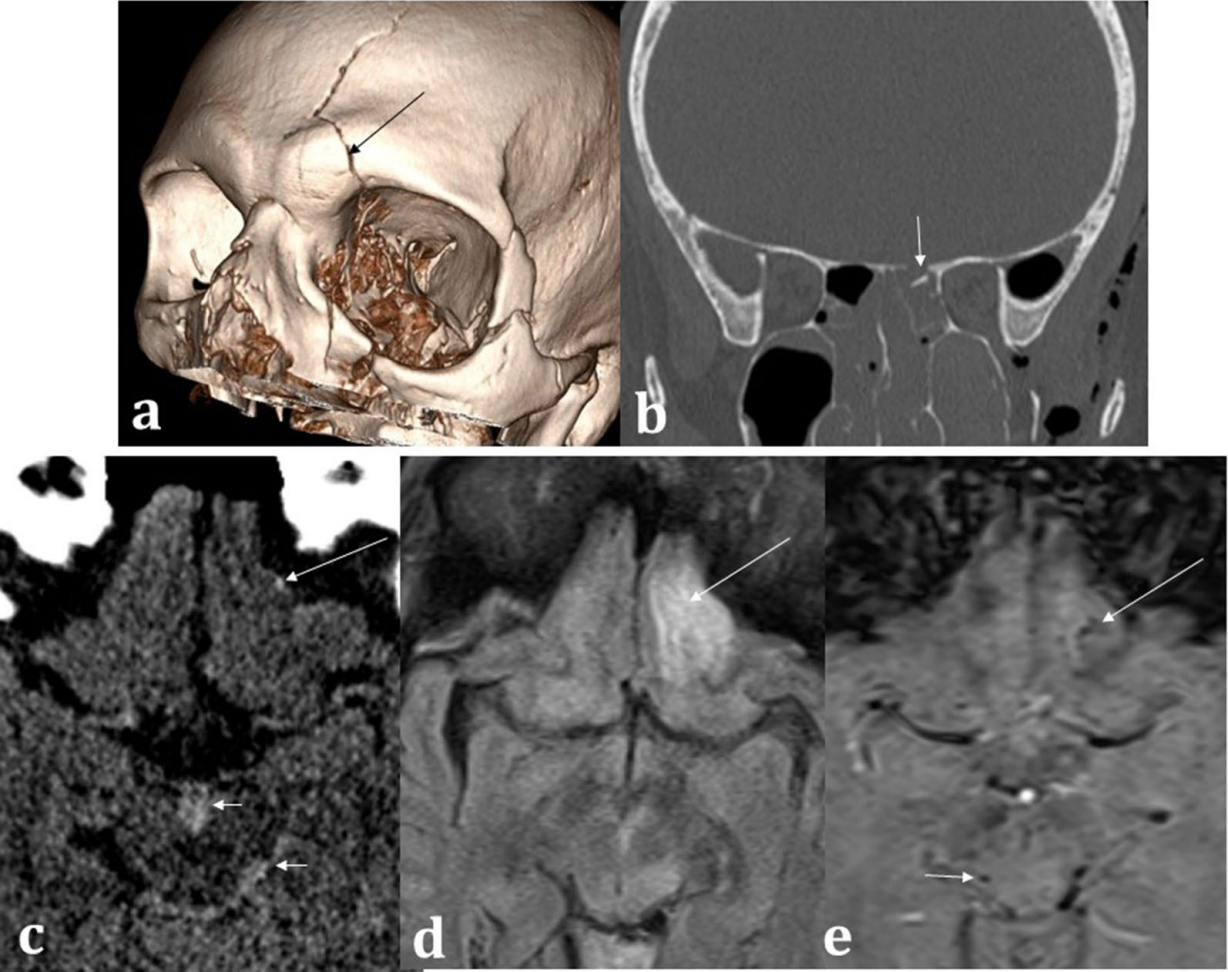
Trauma. 67-year-old male hit by a train. 3D CT reconstruction (**a**) demonstrates left-sided Le Fort III fracture complex and a calvarial fracture involving the frontal bone (arrowed). Coronal bone window CT (**b**) demonstrates an anterior cranial fossa fracture (arrowed) placing the patient at risk of inferior frontal lobe injury. Axial soft tissue window CT (**c**) confirms subtle punctate haemorrhage at the inferior frontal lobe (long arrow) as well as interpeduncular and ambient cistern subarachnoid haemorrhage (short arrows). The MRI axial FLAIR sequence (**d**) demonstrates a corresponding area of abnormal left inferior frontal lobe signal involving the olfactory sulcus (arrowed) with microhaemorrhages on the SWI (**e**) sequence (long arrow). Additional microhaemorrhages within the midbrain are consistent with grade III diffuse axonal injury (short arrows), and the aforementioned cisternal subarachnoid blood is also seen. The patient unfortunately passed away from related traumatic injuries

Trauma is a common cause of anosmia and symptomatic post-traumatic olfactory disturbance is strongly correlated with reduced olfactory bulb volumes [34, 35]. On CT assessment for post-traumatic olfactory symptoms, damage to the olfactory apparatus can be inferred by the presence of frontobasal contusions, whilst fractures of the cribriform plate are suggestive of damage to the fila olfactoria [10]. In some cases, a putative cause cannot be firmly identified on imaging, in which case shearing forces to the fila olfactoria and olfactory nerves at the cribriform plate may be responsible [28]. Acute CT imaging is very useful in identifying trauma-related surgical emphysema, pneumocephalus suggestive of anterior cranial fossa breach, and haemorrhagic or non-haemorrhagic traumatic contusions [39]. Where beam-hardening artefact may impede interpretation of haemorrhage at the anterior cranial fossa, blood sensitive MRI SWI sequences can be performed as an adjunct if necessary (Fig. 14).

### CSF fistulas

The cribriform plate is a common site for acquired CSF fistula formation. A CSF fistula refers to an osteodural defect with consequent fistula formation [40]. This typically presents with CSF rhinorrhoea that can be exacerbated on vagal manoeuvres, and obstructive nasal symptoms with olfactory disturbance. 80% of acquired CSF fistulas are secondary to trauma, most commonly in the setting of anterior skull base fractures [41]. Less commonly, acquired CSF fistulas can arise secondary to iatrogenic causes, tumours, and chronically raised intracranial pressure [42]. Post-traumatic CSF leaks are generally small and can be managed conservatively with bed rest. However even if small, a persistent CSF leak places the patient at increased risk of meningitis and surgery may be required [40]. CT is the first-line to isolate the offending osteodural defect (Fig. 14) and does not require an active leak to be present unlike other imaging techniques (such as radionuclide imaging and CT cisternography) [42]. Though CT can detect even small osseous defects with a sensitivity of 92% and specificity of 100% [40, 42], a dural breach cannot be seen on CT. Isolating the specific site of a CSF fistula therefore becomes particularly problematic when there are numerous osseous defects, for example in the context of complex skull base trauma [42, 43].

**Fig. 15:**
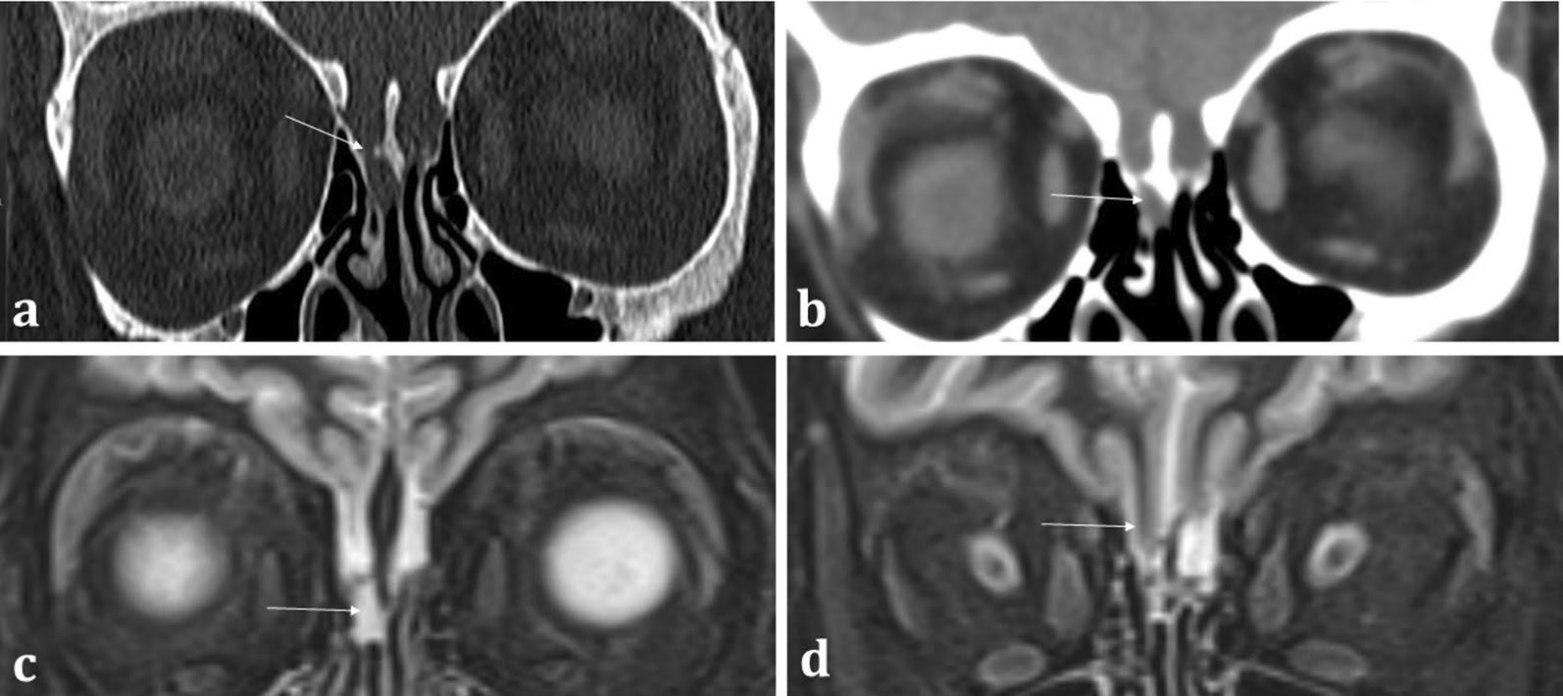
Acquired post-traumatic CSF fistulas in two different patients. Coronal bone (**a**) and soft tissue (**b**) CT images demonstrate a tiny bony defect of the right cribriform plate (arrow, **a**) and abnormal opacification of the right olfactory recess on the soft tissue window (arrow, **b**). The coronal T2WI MRI image of the same patient confirms herniation of fluid intensity CSF into the right olfactory recess consistent with a meningocele (arrow, **c**). For comparison, coronal T2WI MRI image of another patient (**d**) demonstrates herniation of both the gyrus rectus and CSF consistent with a meningoencephalocele (arrow, **d**)

**Fig. 16:**
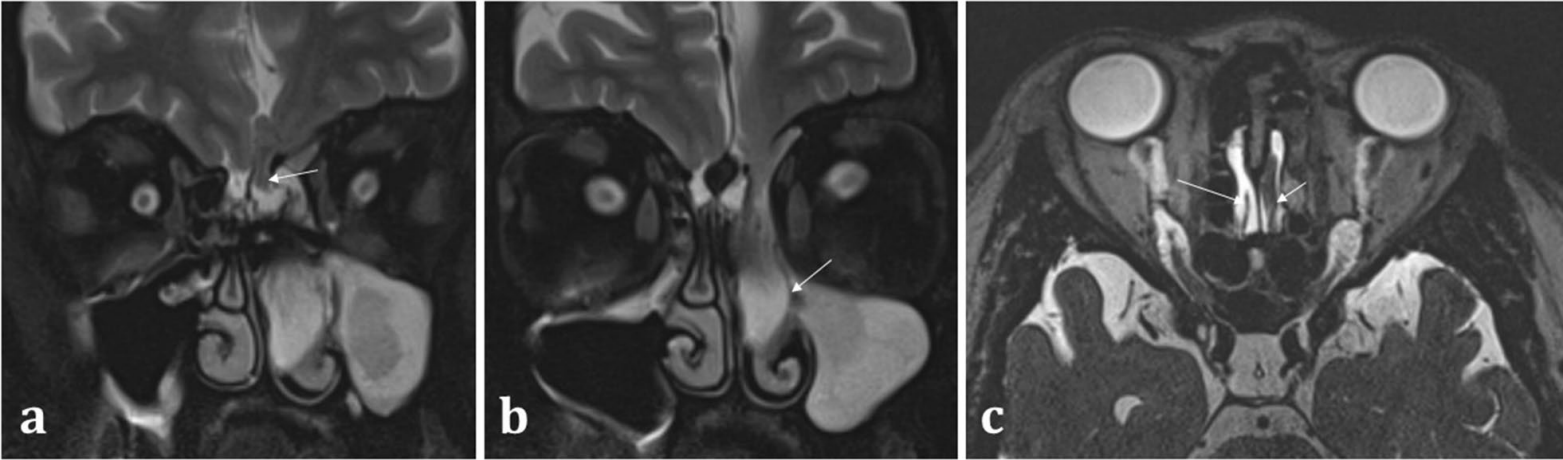
Acquired non-traumatic CSF fistula secondary to idiopathic intracranial hypertension (IIH). 43-year-old female patient diagnosed with IIH at the age of 16 but lost to follow up, presenting with nasal congestion, CSF rhinorrhoea, and blurred vision. Sequential coronal T2W sequences (**a**, **b**) demonstrate a large left meningoencephalocoele herniating through the left olfactory recess (arrow, **a**), extending down into the nasal cavity and obstructing the left osteomeatal complex (arrow, **b**). Heavily T2 weighted CISS sequence (**c**) demonstrates the normal olfactory tract surrounded by CSF on the right (long arrow), and the abnormal herniating brain tissue on the left (short arrow). An “empty sella” with tortuous optic nerves and dilated subarachnoid spaces around the optic nerves are consistent with IIH, which is a well-described cause of acquired CSF fistulas

Correlation with MRI is useful in demonstrating a dural breach, as inferred by high T2 signal CSF (from “meningocele” formation) (Fig. 15). Herniation of brain tissue (“meningoencephalocele”) can also be seen (Figs. 15d, 16), whilst subtle traction-related adjacent encephalomalacia and dural enhancement are both secondary signs of CSF fistula formation [42]. Heavily T2 weighted high spatial resolution CISS imaging provides optimal contrast between the CSF and the neighbouring brain and skull base and is a sensitive sequence for the detection of CSF fistulas [43]. However, CSF fistulas must be differentiated from sinusitis and other fluid intensity substances such as nasal secretions [42, 43]. Accurate localisation of the defect can direct endoscopic surgical repair, which carries reduced morbidity and is therefore being increasingly adopted over open repair [40].

### Skull base and dural lesions

**Fig. 17:**
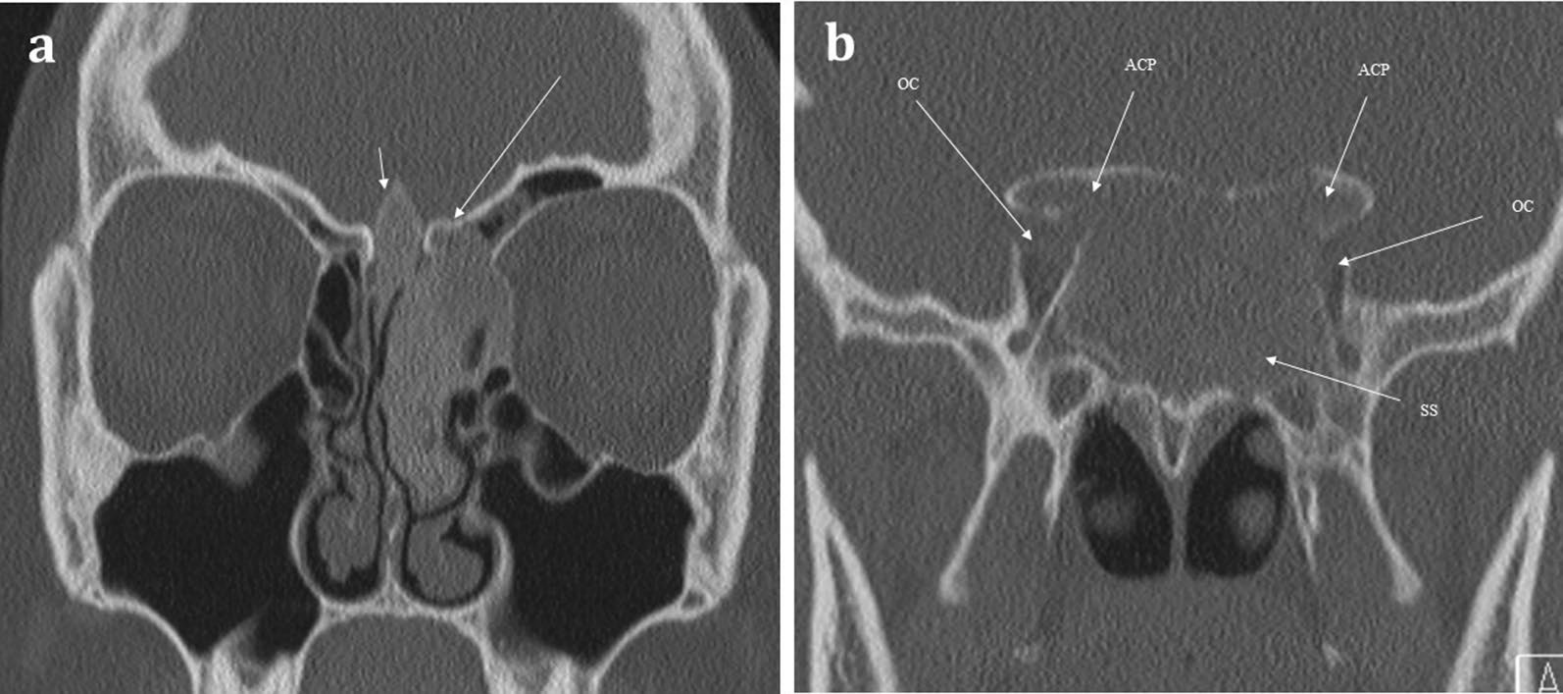
Fibrous Dysplasia. 24-year-old female patient with history of diabetes and café au lait spots presenting with long standing anosmia and nasal congestion, and acute left sided visual loss. Coronal CT images showing expansile multifocal osseous lesions with typical internal ground-glass matrix, involving the left cribriform plate (long arrow, **a**) and crista galli (short arrow, **a**). This is causing remodelling of the olfactory fossae and is affecting the olfactory pathway. There are similar expansile lesions affecting the anterior clinoid processes (ACP) and the sphenoid sinus (SS) which causes compromise of both optic nerve canals (OC), worse on the left—this accounts for the visual loss in the left eye. The patient was diagnosed with McCune–Albright syndrome (polyostotic fibrous dysplasia with cutaneous and endocrine abnormalities), and surgical decompression of the left optic canal was performed

Intrinsic lesions of the anterior skull base can affect the olfactory epithelium, olfactory bulbs, and tracts. This includes osseous abnormalities such as Paget’s disease or fibrous dysplasia [11, 44]. Both of these pathologies produce expansile lesions that can encroach upon and obstruct the nasal cavity or anterior cranial fossa (Fig. 17) [16].

### Neurodegenerative conditions

The traditional role of imaging in neurodegenerative disease is to map out the affected anatomical regions, assess brain volumes, and identify and exclude other competing or contributing pathologies. These include normal pressure hydrocephalus, ischaemia, or high burden of small vessel disease [2].

Olfactory disturbances can precede the onset of other symptoms in neurodegenerative diseases such as idiopathic Parkinson’s disease (IPD) and Alzheimer’s disease (AD) [6, 28]. Olfactory deficits, however, are also commonly seen as part of the normal ageing process [8]. Therefore, an emerging challenge for radiologists is to employ imaging techniques to help differentiate age-related olfactory deficits from an early manifestation of neurodegenerative disease, to facilitate earlier diagnosis.

### Parkinson’s disease

Olfactory dysfunction in IPD is very common, occurring with the same frequency as resting tremor [45], and can precede motor symptom onset [46]. A marked loss of olfaction is interestingly not seen in benign essential tremor [47] or atypical Parkinsonism [48]. As such, olfactory dysfunction has been proposed as both a screening for IPD [49], and to clinically distinguish IPD from other competing differentials [48, 50, 51]. This is particularly relevant for atypical Parkinsonism, where the diagnosis can be challenging, with characteristic imaging findings only seen late in the disease [52].

Autopsy specimens have identified Lewy bodies- the pathological hallmark of the disease- within both the peripheral olfactory bulb and central cortical regions of the olfactory system [49]. However, there is lack of concordance in the literature as to whether olfactory bulb volumes are significantly reduced in IPD compared to control subjects [49, 53, 54], as it has been well-described that olfactory bulb volumes normally decrease with age [8]. Nevertheless, on a microstructural scale, diffusion tensor imaging can reveal increased diffusivity within the olfactory bulbs indicative of cellular degeneration in early IPD [55]. Moreover, MRI measurements of volume loss within the right piriform cortex in early IPD are correlated significantly with extent of olfactory dysfunction, when compared to age-matched controls [56].

### Alzheimer’s disease

Olfactory dysfunction can occur early in AD and precede the onset of dementia [57]. This corresponds with the histopathological findings of neurofibrillary tangles (NFTs) within both the olfactory bulbs and tracts and centrally within the primary olfactory cortex [58]. This distribution of NFTs within the olfactory system is also seen in individuals with early AD [59]. Though the density of NFTs correlates with more severe disease, NFTs can also be present within the olfactory system as part of normal ageing [59]. Nevertheless, in patients with early AD, it has been found that functional MRI activity within the primary olfactory cortex [60] and the volume of olfactory bulb and tract on MRI [61] are significantly decreased when compared to age-matched control subjects.

## Conclusion

The olfactory pathway represents a complex interplay between peripheral and central elements and is susceptible to myriad of different pathologies. Appropriate CT, MRI, or combined modality imaging can help to differentiate benign from malignant conditions, better characterise the nature of a lesion or disease process, and isolate the specific anatomical elements of the olfactory system that are involved.

## Data Availability

Not applicable.

## Abbreviations

AB: Amygdaloid body
ACP: Anterior clinoid process
AD: Alzheimer's disease
ADC: Apparent diffusion coefficient
CG: Crista galli
CISS: Constructive interference in steady state
COVID-19: Coronavirus disease 2019
CP: Cribriform plate
CSF: Cerebrospinal fluid
CSF: Collateral sulcus
CT: Computed tomography
DWI: Diffusion weighted imaging
EAC: Ethmoid air cells
EC: Entorhinal cortex
FE: Fovea ethmoidalis
FLAIR: Fluid-attenuated inversion recovery
GPA: Granulomatosis with polyangiitis
GR: Gyrus rectus
HPV: Human papillomavirus
IIH: Idiopathic intracranial hypertension
IPD: Idiopathic Parkinson's disease
ITG: Inferior temporal gyrus
LL: Lateral lamella
LV: Lateral ventricles
MRI: Magnetic resonance imaging
MS: Maxillary sinus
MTG: Middle temporal gyrus
NFT: Neurofibrillary tangles
OB: Olfactory bulb
OC: Optic nerve canals
OG: Orbital gyrus
OR: Olfactory recess
OS: Olfactory sulcus
PC: Piriform cortex
SC: Sinonasal compartment
SCC: Squamous cell carcinoma
SS: Sphenoid sinus
SWI: Susceptibility weighted imaging
T: Tesla
T1WI: T1 weighted imaging
T2WI: T2 weighted imaging
